# Association between gender inequality index and child mortality rates: a cross-national study of 138 countries

**DOI:** 10.1186/s12889-015-1449-3

**Published:** 2015-03-09

**Authors:** Ethel Mary Brinda, Anto P Rajkumar, Ulrika Enemark

**Affiliations:** Department of Public Health, Section for Health Promotion and Health Services, Aarhus University, Aarhus, 8000 Denmark; Translational Neuropsychiatry Unit, Aarhus University Hospital, Risskov, 8240 Denmark; Department of Biomedicine, Aarhus University, Aarhus, 8000 Denmark

**Keywords:** Child mortality, Ecological study, Gender, Women, Empowerment

## Abstract

**Background:**

Gender inequality weakens maternal health and harms children through many direct and indirect pathways. Allied biological disadvantage and psychosocial adversities challenge the survival of children of both genders. United Nations Development Programme (UNDP) has recently developed a Gender Inequality Index to measure the multidimensional nature of gender inequality. The global impact of Gender Inequality Index on the child mortality rates remains uncertain.

**Methods:**

We employed an ecological study to investigate the association between child mortality rates and Gender Inequality Indices of 138 countries for which UNDP has published the Gender Inequality Index. Data on child mortality rates and on potential confounders, such as, per capita gross domestic product and immunization coverage, were obtained from the official World Health Organization and World Bank sources. We employed multivariate non-parametric robust regression models to study the relationship between these variables.

**Results:**

Women in low and middle income countries (LMICs) suffer significantly more gender inequality (p < 0.001). Gender Inequality Index (GII) was positively associated with neonatal (β = 53.85; 95% CI 41.61-64.09), infant (β = 70.28; 95% CI 51.93-88.64) and under five mortality rates (β = 68.14; 95% CI 49.71-86.58), after adjusting for the effects of potential confounders (p < 0.001).

**Conclusions:**

We have documented statistically significant positive associations between GII and child mortality rates. Our results suggest that the initiatives to curtail child mortality rates should extend beyond medical interventions and should prioritize women’s rights and autonomy. We discuss major pathways connecting gender inequality and child mortality. We present the socio-economic problems, which sustain higher gender inequality and child mortality in LMICs. We further discuss the potential solutions pertinent to LMICs. Dissipating gender barriers and focusing on social well-being of women may augment the survival of children of both genders.

## Background

Gender is a multidimensional social construct, with distinct roles attributed to men and women in a specific society [1]. Gender based stereotypes leads to inequalities in access to fundamental human rights including nutrition, education, employment, health care, autonomy and freedom. Increase in female morbidity and mortality through feticide, infanticide [2], genital mutilation [3], physical as well as sexual violence, contribute to millions of *missing women* around the globe [4]. The consequences of gender inequality are extensive. Beyond harming the health of women, gender inequality hinder global economic growth and overall social development [5].

Gender equality is a vital strategic component of the United Nations (UN) Millennium Development Goals, to reduce the rates of under-five mortality, maternal mortality as well as HIV infections and to promote universal primary education. United Nations Children’s Fund recognises that empowerment of women has major influence on child survival and wellbeing [6]. Recent World Development Report indicates gender equality as an instrument to enhance overall economic productivity of a society and positive health outcomes of children [7]. However, systematic research on gender inequality and on its health implications, remains sparse, because of the difficulties in measuring the multidimensional nature of gender inequality [8]. In 2008, United Nations Development Programme (UNDP) developed a *Gender Inequality Index* (GII) which is currently available for 138 countries [9]. GII is a composite measure, including three dimensions, reproductive health, empowerment, and labour participation of women [9,10]. These dimensions are derived from five major indicators, including percentage of higher (secondary level and above) education attainment by women, parliamentary representation of women, labour force participation by women, maternal mortality rate, and adolescent fertility rate.

Gender inequality fuels maternal under-nutrition, and increases the incidence of low birth weight (LBW) babies [11] and of malnutrition of children of both genders [12,13]. Such malnutrition and associated infectious diseases challenge the survival of children [14]. This pathway is further reinforced by the link between maternal education and child mortality [15]. Various studies have envisaged the association between violence against women and child mortality rates [11] in rural areas. However, the associations between GII and official child mortality rates, around the globe, remains unknown. As child mortality rates are major indicators of overall health and development of populations, understanding the association between GII and child mortality rates will have broad implications beyond the health of children. Major macroeconomic indices such as economic inequality (Gini index) and Gross National Income [16] as well as health service indicators such as immunization coverage [17] influence child mortality rates, and may also be related to GII. As pertinent studies evaluating the confounding effects of these major macroeconomic and health service variables are currently not available, we aimed to study the association between GII and child mortality rates, while adjusting for these potential confounders.

## Methods

### Study design

We employed an ecological study design to study the association between GII and various child mortality rates among 138 countries.

### Data collection

GII was available for 138 countries at the time of this study [10]. It ranges from zero to one, with higher scores indicating more gender inequality. We obtained the following mortality data of those 138 countries: (i) Infant mortality rate (IMR), (ii) neonatal mortality rate (NMR), (iii) under-five mortality rate (U5MR), (iv) female U5MR, (v) rural U5MR, (vi) U5MR due to HIV/AIDS, (vii) U5MR due to pneumonia and (viii) U5MR due to diarrhoea, from the official global database, WHO statistical Information system (WHOSIS) [18]. We accessed the data on immunization coverage rates among one year old children from WHOSIS and the per capita Gross Domestic Product (per capita GDP) of these countries were retrieved from World Bank’s economy indicators [19]. Finally, we retrieved the economic inequality index (Gini coefficient) of 129 of these countries from Central Intelligence Agency’s global database [20]. All data, used in this study, are openly available online.

### Statistical analysis

We initially analysed the study variables using descriptive statistics and checked whether all continuous variables followed Gaussian distribution by one sample Kolmogorov-Smirnov tests. The correlation between the child mortality rates and GII were initially assessed by spearman’s rank order correlation. Then, we studied the association between GII and various child mortality rates with robust regression models, using STATA *rreg* command. Robust regression models are valid to reduce the influence of the non-normal residuals and they are less sensitive to outliers. They initially execute ordinary least squares regression to identify Cook’s D value for each observation. After highly influential outliers are set aside, iteration process begins with calculation of weights using Huber and Tukey function to give less importance to the observations with larger residuals. After several iterations, weighted least squares are performed to estimate regression coefficients. We performed multivariate non-parametric robust regression models to adjust for the potential confounders. Coefficients of determination (R^2^) for the robust regression models were calculated using STATA *rregfit* command. All statistical analyses were performed using statistical software STATA 12.1.

## Results

### Participant countries

We compiled the complete data of 138 countries, which included 27 low income, 38 low middle income, 30 upper middle income and 43 high income countries. They span over all six WHO regions, including African (31), American (26), East Mediterranean (17), European (44), South East Asian (7) and Western Pacific (13) regions. 6.6 billion People live in these countries, which forms more than 92% of the world population at present. The countries included in this study (N = 138) did not differ significantly from the countries, for which GII data have not been published (N = 55), among their per capita Gross Domestic Product (GDP) (Mann–Whitney U = 3283.5; p = 0.14), immunization coverage rates (U = 3748; p = 0.89), IMR (U = 3233.5; p = 0.10), NMR (U = 3160.5; p = 0.07) and U5MR (U = 3252.0; p = 0.12).

### Nature of GII and under-five mortality rates

The GII values ranged from 0.174 (in Netherlands) to 0.835 (in Yemen). The mean value of GII of 138 countries was 0.53 (SD 0.16; median = 0.57). Women living in Low and middle income countries (LMICs) suffer significantly more gender inequality than those living in high income countries (Kruskall Wallis χ^2^ = 90.1; df = 3; p < 0.001). There were significant inverse correlations between GII and per capita GDP (Spearman ρ = −0.82; p < 0.001) as well as between GII and Immunization coverage (Spearman ρ = −0.52; p < 0.001). GII and the economic inequality index had significant positive correlation (Spearman ρ = 0.56; p < 0.001).

The mean value of U5MR of 138 countries was 42 per 1000 live births (SD 49; median = 21). The U5MR was significantly higher in LMICs than in high income countries (Kruskall Wallis χ^2^ = 104.1; df = 3; p < 0.001). Significant negative correlations were present between U5MR and per capita GDP (Spearman ρ = −0.89; p < 0.001) as well as between U5MR and Immunization coverage (Spearman ρ = −0.55; p < 0.001).

### Association between GII and child mortality rates

**Figure 1:**
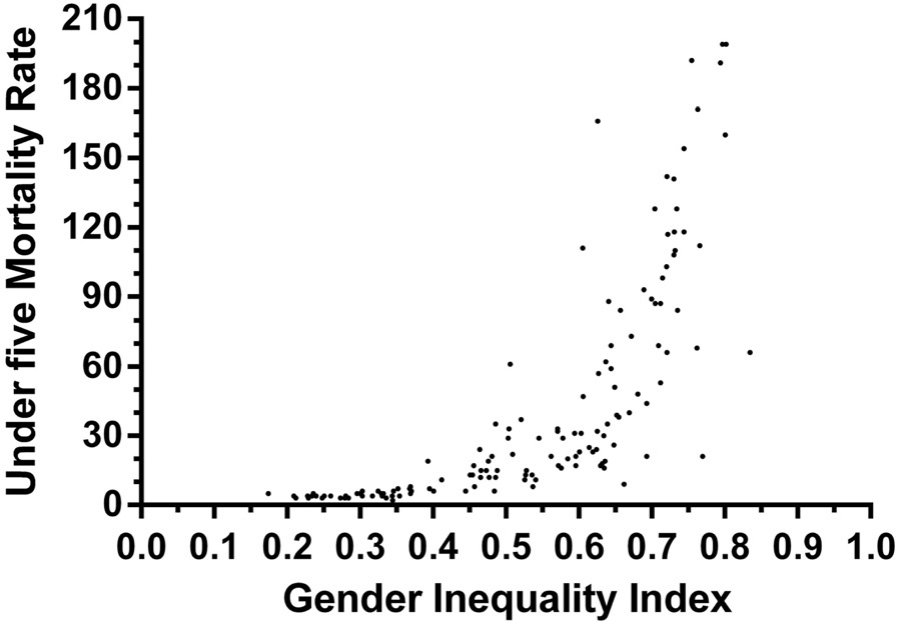
**Association between gender inequality index (GII) and under five child mortality rates (per 1000 live births) in 138 countries.**

GII had significant positive correlation with NMR (Spearman ρ = 0.98; p < 0.001), IMR (Spearman ρ = 0.99; p < 0.001) and U5MR (Spearman ρ = 0.91; p < 0.001). We present the bivariate and multivariate robust regression models for the association between GII and child mortality rates in Table 1. We present the scatter plot between the GII and U5MR of 138 countries as Figure 1. Various child mortality rates had significant association with GII after adjusting for per capita GDP and immunization coverage. These multivariate models including GII could explain 57% of variability in NMR (R^2^ = 0.57), 43% of variability in IMR (R^2^ = 0.43) and 32% of variability in U5MR (R^2^ = 0.32), around the globe. We calculated the female to male mortality ratios by dividing female U5MR by male U5MR. This ratio was also significantly associated with increasing GII (β = 26.8; 95% CI = 12.8-40.8; p <0.001), after adjusting for the confounders.

**Table 1:** **Association between gender inequality index (GII) and child mortality rates** ^**a**^ **in 138 countries**

**Dependent variable**	**Bivariate statistics** ^**b**^			**Multivariate statistics** ^**c**^		
	**β (95% CI)**	**t**	**p value**	**β** ^**d**^ **(95% CI)**	**t**	**p value**
Neonatal mortality rate	61.76 (54.25-69.26)	16.27	**<0.001**	53.85 (41.61-64.09)	9.30	**<0.001**
Infant mortality rate	109.03 (93.35-124.71)	13.75	**<0.001**	70.28 (51.93-88.64)	7.57	**<0.001**
Female under-five mortality rate	115.54 (96.68-134.40)	12.12	**<0.001**	61.88 (44.48-79.27)	7.04	**<0.001**
Rural under-five mortality rate^e^	419.90 (302.20-537.60)	7.13	**<0.001**	290.43 (145.64-435.22)	4.01	**<0.001**
Under-five mortality due to pneumonia	33.37 (27.87-38.87)	12.00	**<0.001**	21.70 (13.20-30.19)	5.05	**<0.001**
Under-five mortality due to diarrhoea	30.62 (26.35-34.90)	14.17	**<0.001**	28.12 (21.58-34.67)	8.50	**<0.001**
Under-five mortality due to HIV	0.74 (0.35-1.13)	3.77	**<0.001**	0.69 (0.04-1.34)	2.11	**0.03**
Under-five mortality rate	107.09 (90.58-123.59)	12.83	**<0.001**	68.14 (49.71-86.58)	7.31	**<0.001**

^a^per 1000 live births; ^b^Robust regression models with GII as independent variable; ^c^Multiple robust regression models with GII as independent variable and Per capita Gross Domestic Product (in US$) as well as immunization coverage among children as covariates; ^d^Regression coefficient of GII, adjusted for the effects of above listed covariates; ^e^Data are available only from 82 countries.

## Discussion

This study confirmed that there were significant positive associations between gender inequality index and neonatal, infant as well as under-five child mortality rates, after adjusting for the effects of major economic and health service variables.

### Strengths and limitations

GII is a relatively new index, and this study is the first of its kind to analyse the global association between GII and child mortality rates. Strengths of our study were including complete and most recent data available from the official sources, and employing multivariate statistics as well as non-parametric models. As the presence of influential outliers among social and health indices is common in global data-bases, appropriate non-parametric robust regression models are necessary to investigate such data. We should acknowledge the following limitations. Firstly, gender inequality is a complex multidimensional phenomenon, and the definition of GII is still evolving [8]. Secondly, all ecological studies have a potential limitation of *ecological fallacy*, which is an association observed between the study variables on an aggregate level, not necessarily representing the association that exists at an individual level. Thirdly, causal associations can only be speculated from this cross-sectional study design. However, the longitudinal data on GII were not available at the time of these analyses. Fourthly, there may be a possibility of some LMICs under-reporting their child mortality rates, and not regularly updating their maternal mortality rates [21]. Furthermore, we did not include many potential confounding variables in our multiple robust regression models, because their availability is limited in LMICs, or there are concerns related to multi-collinearity. We did not adjust for the low birth weight rates, because they are involved in the causal pathway between GII and childhood mortality rates.

### Pathways connecting gender inequality and child mortality

Gender inequality harms children during antenatal, perinatal, postnatal periods and during further development. GII may cause child mortality in one of the following direct pathways,

1. Female infanticide and female circumcision contribute to a small but ominous proportion of child mortality [2].
2. LMICs with high GII have higher prevalence of maternal under-nutrition [22]. Consequent intrauterine growth retardation leads to more LBW babies and biologically disadvantaged children, who are vulnerable to infectious diseases. Our results have supported such positive association between GII and U5MR due to infectious diseases.
3. Maternal exposure to domestic violence increases the risk for LBW and preterm births [11]. Witnessing domestic violence against their mothers brings up more psychosocially disadvantaged children.
4. Reduction of 4.2 million deaths of children below five years, between the years 1970 and 2009, was attributed to the better educational attainment of women [15]. Women with inequitable access to education cannot aid the survival of their children by appropriate feeding and preventive health practices.
5. Our findings confirmed the positive association between GII and U5MR due to HIV and AIDS. Gender violence [23] increases the risk of women acquiring HIV infection and other sexually transmitted diseases. Lack of autonomy hinders women equitably accessing health education and preventive as well as curative health services [24,25] to prevent transmission of disease to their children.
6. Women’s control over household economy can help reducing child mortality [26]. Mothers, lacking economic autonomy, cannot guide their household finances towards better nutrition and health of their children [27].
7. Prevalence of malnutrition is higher among girls than boys in many countries [22]. Such deprivation and the negative social environment compromise the survival of female children.

There are numerous indirect pathways connecting gender inequality and child mortality [22].

### Socioeconomic perspectives of GII

Gender inequality is connected with many social evils and makes them heritable across generations. As a detailed discussion of all negative social consequences of GII is beyond the scope of this short report, we briefly discuss three germane social issues, which largely influence child mortality rates, especially in LMICs. First, *son preference* is widely prevalent in many societies and is associated with high female perinatal as well as infant mortality rates [28,29]. Unwanted girls, born to multiparous women without any living sons, have significantly less odds to survive or they grow up in adverse psychosocial circumstances [30]. Secondly, *Dowry* is a social practice, in which a girl’s parents are forced to offer material riches to groom’s family to conduct her marriage [31]. Female suicides and homicides due to dowry harassment are not uncommon in LMICs. This social evil incites the *daughter aversion,* and many female infanticides [32]. Thirdly, *Mathew effect*, explains the persistence of high child mortality rates in LMICs with poor macroeconomic indicators [33,34]. Our results have confirmed the significant inverse correlation between per capita GDP and U5MR and have indicated the role of GII in this vicious cycle. The relationship between per capita GDP and GII can be bidirectional [35]. Countries, where women have higher educational attainment and more labour participation, prosper economically and attain further reductions in their child mortality rates. In contrast, high GII keeps countries poor and sustains their child mortality rates.

### Why does gender inequality persist in LMICs?

Despite the persistent efforts to curtail gender injustice by the Governments, non-governmental organizations (NGO) and feminist movements over many decades, gender equality remains as a distant ideal in many LMICs. Our results showed that LMICs have significantly higher GII and U5MR than the high income countries. Success of feminism in high income countries has not been replicated in LMICs, due to the following barriers,

1. Gender stereotypes are culturally ingrained and are sanctified by religions. Gender initiatives are often viewed as threats to local culture, tradition and religious beliefs.
2. Despite improving the female literacy rates over the past decades, drop-out rates from secondary level education and above remain persistently higher among girls in LMICs [36]. Recent industrialization in LMICs reduces the employment opportunities of girls, who lack higher education.
3. Our results showed that GII is positively correlated with economic inequality. Gender equality is one of the many basic human rights denied to poorer sections of the society by the prevailing high economic inequality in LMICs. Feminist ideology has reached mostly the affluent and remains alien to many poor rural women in LMICs.
4. Patriarchal family systems and property inheritance sustain son preference and dowry customs. Many women, beyond their middle ages, get attuned to their gender roles and collude with authoritarian men to ensure subordination of younger women in their families.
5. Increasing need for out-of-pocket health expenditures causes inequitable access to health services [37]. Many poor and less educated women avoid utilizing health services, especially prevention services, and worsen their health standards, due to the fear of catastrophic health expenditures [38].
6. Narrow medical perspectives often reduce gender equality to primary care reproductive health and invest their resources mostly on curative medical interventions [39].
7. Predominant business interests lure the media to endorse the gender stereotypes of patriarchal societies.

### Removing barriers to gender equality

As both sexes are innately equal, gender equality need not be considered as a far-off ideal. Our results suggest the following to remove the man-made barriers against gender equality and to aid the survival of more children,

1. Patriarchal societies often concern more about the well-being of their progeny than that of their women. The relationship between their gender inequality and the survival of their children should be highlighted in every possible way to make them feel the need for a social change.
2. GII is positively correlated with per capita GDP. Gender equality cannot be achieved in isolation in a starving society. Policies envisioning poor national economies to be stronger and be independent of external aids are essential to progress gender equality.
3. GII is positively correlated with economic inequality index. Inequitable economic growth can do more harm than good for gender equality. Poor women, who lack skilled education, rely on agricultural labour and small businesses for their autonomy. Their interests should be preserved during the current wave of capitalist boom in LMICs.
4. GII is inversely correlated with immunization coverage. Preventive, let alone curative, medical interventions have limited success to curtail high child mortality rates in LMICs, where broader social objectives are often less prioritized. Existing health services should join their hands with social initiatives prioritizing women autonomy to achieve desired health indicators.
5. Rise in female literacy is not accompanied by corresponding reduction of gender inequality and gender violence [40] in LMICs. Industrialization of societies demands more skills, than the ability to read and write, to lead an autonomous life. Education policies for women should emphasize developing skills, rather than imparting more knowledge. Governments should realize that spending on higher education of women is a wise investment to accelerate their economic growth [35], to prevent more child mortality and to reduce their expense on curative health services [15]. Ensuring equitable access to higher education and investigating the determinants of female higher education dropout rates are essential.
6. Feminist ideology should be tailored to the needs of individual countries [41]. Conflicts with the local culture and religion should be discussed publicly [39] and be resolved with the help of shared motives for the welfare of involved communities. Feminist movements should move their urban bases in LMICs closer to the poor rural women, who need their services more. Striving to gain the co-operation of existing social networks and integrating themselves with the poorer sections of the societies will aid to realize the vision of feminists in LMICs.
7. Need for out-of-pocket health expenditure connects economic inequality, gender inequality and the inequities in the delivery of health care to poor women in LMICs. Minimizing out-of-pocket health expenditures and improving the standards of existing public health services will ensure better maternal health and survival of children.

## Conclusions

This study has documented significant positive associations between GII and child mortality rates. Our findings suggest that the initiatives to curtail child mortality rates should extend beyond medical interventions, and should prioritize women’s rights as well as autonomy. Holistic multidimensional initiatives, which focus on social well-being of women and dissipate gender barriers, are the need of the hour to augment the survival of children of both genders.
